# A multi-country survey of the socio-demographic factors associated with adherence to COVID-19 preventive measures during the first wave of the COVID-19 pandemic

**DOI:** 10.1186/s12889-023-16279-2

**Published:** 2023-07-24

**Authors:** Morenike Oluwatoyin Folayan, Roberto Ariel Abeldaño Zuñiga, Jorma I. Virtanen, Oliver C. Ezechi, Muhammad Abrar Yousaf, Mohammed Jafer, Ala’a B. Al-Tammemi, Passent Ellakany, Eshrat Ara, Martin Amogre Ayanore, Balgis Gaffar, Nourhan M. Aly, Ifeoma Idigbe, Joanne Lusher, Maha El Tantawi, Annie L. Nguyen

**Affiliations:** 1 Mental Health and Wellness Study Group, Ile-Ife, Nigeria; 2 Department of Child Dental Health, Obafemi Awolowo University, Ile-Ife, Nigeria; 3 Centre for Social Data Science, Faculty of Social Sciences, University of Helsinki, Helsinki, Finland; 4 Postgraduate Department, University of Sierra Sur, Oaxaca, Mexico; 5 Faculty of Medicine, University of Turku, Turku, Finland; 6 The Centre for Reproductive and Population Health Studies, Nigerian Institute of Medical Research, Yaba, Lagos, Nigeria; 7 Department of Biology, Faculty of Science and Technology, Virtual Univesity of Pakistan, Lahore, Pakistan; 8 Dental Public Health Division, Faculty of Dentistry, Jazan University, Dammam, Saudi Arabia; 9 Applied Science Research Center, Applied Science Private University, Amman, Jordan; 10 Migration Health Division, International Organization for Migration, Amman, Jordan; 11 Department of Substitutive Dental SciencesCollege of Dentistry, Imam Abdulrahman Bin Faisal University, Dammam, Saudi Arabia; 12 Department of Psychology, Governemnt College for Women, MA Road, Srinagar, J&K India; 13 Department of Health Policy Planning and Management, Fred N. Binka School of Public Health, University of Health and Allied Sciences, Ho, Ghana; 14 Department of Preventive Dental Sciences, College of Dentistry, Imam Abdulrahman Bin Faisal University, Dammam, Saudi Arabia; 15 Department of Pediatric Dentistry and Dental Public Health, Faculty of Dentistry, Alexandria University, Alexandria, Egypt; 16 Clinical Sciences Department, Nigerian Institute of Medical Research, Lagos, Nigeria; 17 Provost’s Group, Regent’s University, London, UK; 18 Department of Pediatric Dentistry and Dental Public Health, Faculty of Dentistry, Alexandria University, Alexandria, Egypt; 19 Department of Family Medicine, Keck School of Medicine, University of Southern California, Los Angeles, CA USA

**Keywords:** COVID-19, Health behaviour, Prevention, Social determinants of health

## Abstract

**Background:**

Health behaviours are influenced by individual characteristics including age, gender, education and economic level. This study aimed to assess the associations between individual-level determinants and adherence to COVID-19 preventive measures.

**Methods:**

We performed secondary analyses of international data collected using an online survey during the first wave of the COVID-19 pandemic between June and December 2020. The dependent variables were self-reported adherence to COVID-19 preventive measures (wearing of face masks, frequent washing/sanitizing of hands, physical distancing, working remotely). The independent variables were age, sex at birth (female vs male), having a chronic disease related elevated risk for severe COVID-19 (none/little, might be at increased risk, at increased risk), educational level completed (no formal education, primary, secondary vs college/university) and employment status (retiree, students, not employed vs employed). Four multivariate logistic regression analyses were conducted to determine the associations between the dependent variables and independent variables. Interaction terms with country-income level were tested in regressions to explore its moderating effect.

**Results:**

Out of 16,866 respondents, 12,634 (74.9%) wore masks or face coverings, 12,336 (73.1%) washed or sanitized their hands frequently, 11,464 (68.0%) reported adherence to physical distancing and 5,646 (33.5%) worked remotely. In adjusted analyses, increased age, college/university education, employment, and having risks for severe COVID-19 were associated with significantly higher odds of adhering to COVID-19 preventive measures. Retirees and students had lower odds of adhering to COVID-19 prevention measures than employed individuals. Males had significantly lower odds of wearing face masks (AOR: 0.901), frequent washing/sanitizing hands (AOR: 0.774) and working remotely (AOR: 0.875) compared to females. Country-income level generally moderated the above relationships such that the associations disappeared in lower income countries.

**Conclusion:**

The study findings suggest that the individual socio-demographic factors—age, sex, employment status, education status and having a chronic disease – influence adherence to COVID-19 preventive measures. Findings further reiterate the need for health education and health promotion campaigns on preventive health measures to focus on subpopulations, such as younger males, students and retirees, that require targeted or unique messaging.

## Introduction

Adherence to COVID-19 preventive measures is crucial for global containment efforts. During the initial wave of the pandemic, preventive measures such as face mask usage, handwashing, physical distancing, and avoiding crowded places were advocated to minimize the spread of the virus [1]. Non-adherence to these measures poses a significant public concern and hinders the control of the virus [2], while higher adherence is associated with early containment of the disease [3].

Previous studies have highlighted differences in adherence to COVID-19 preventive measures based on sociodemographic characteristics [4]. These characteristics reflect the influence of broader social and economic factors on compliance with restrictive measures [5]. Age, sex, education level, and chronic disease status have been reported as factors associated with adherence to COVID-19 prevention [5–7]. For instance, studies have shown that females [8–10], older individuals [11, 12], those with higher education [13, 14], employed individuals [15], and individuals with chronic diseases [16] demonstrate greater adherence. However, these findings are often specific to individual countries, and there is a lack of macro-analyses exploring how these relationships may vary across countries.

Country income level is a macro-level social determinant of health with policy implications. In low-income countries, the out-of-pocket cost of healthcare is high, limiting individuals' access to medical care [17–19]. This leads to delayed access to appropriate healthcare, including proper diagnoses [20]. Additionally, households in low-income countries tend to spend less on preventive healthcare, which can be attributed to limited access to healthcare providers and preventive services [21]. Given the variations in healthcare-seeking behaviors based on country income level, differences in the use of COVID-19 preventive measures may also exist.

This study adopts the Health Belief Model [22, 23], which suggests that individuals' adoption of recommended health actions is influenced by their perceived benefits of health information and their self-efficacy in engaging in those actions [24]. Confidence in disease prevention [25, 26] is influenced by age, sex, education level, and the presence of chronic conditions. Previous research has shown that confidence in disease prevention decreases with age and is associated with lower health literacy and income [27]. Females [28–30], individuals with chronic diseases [31], and those with higher education [32] tend to have more confidence in their ability to engage in disease prevention actions.

Health efficacy is influenced by social resources such as education, socioeconomic status, and income, resulting in a social gradient in health, where higher levels of social resources correspond to better health outcomes [33, 34]. Country-level income also contributes to this social gradient. Social inequalities can impact the adoption and adherence to COVID-19 preventive measures, and understanding the role of social factors in adherence can inform interventions to mitigate unfavorable social gradients and enhance pandemic control.

This study aims to examine the associations between age, sex, employment status, education status, and the presence of chronic diseases related to elevated risk for severe COVID-19 and adherence to preventive measures during the initial wave of the pandemic. Additionally, we investigate how country-level income moderates these associations. Our hypothesis is that younger age, males, individuals with lower educational status, and those without chronic diseases related to elevated risk for severe COVID-19 would be less likely to adhere to preventive measures, and that country income levels would influence these associations.

## Methods

### Ethical considerations

The primary study received ethical approval from the Human Research Ethics Committee at the Institute of Public Health of the Obafemi Awolowo University Ile-Ife, Nigeria (HREC No: IPHOAU/12/1557). Additional ethical approvals were obtained from Brazil (CAAE N° 38,423,820.2.0000.0010), India (D-1791-uz and D-1790-uz), Saudi Arabia (CODJU-2006F), and the United Kingdom (13,283/10570).

### Study design and study population

The present study involved a secondary analysis of data derived from an international cross-sectional study. The study recruited participants from 152 countries using an online survey platform (Survey Monkey, Momentive Inc.: San Mateo, CA, USA) between July and December 2020, which was conducted during the initial phase of the COVID-19 pandemic. Eligibility for study participation was open to individuals aged 18 years and older, and written consent was obtained from all participants. No specific exclusion criteria were applied.

### Sample size

The primary study's sample size was determined by considering the highest global prevalence of a mental health disorder in 2019, specifically anxiety disorder (3.94%) [35]. A desired precision estimate of 0.05 and a confidence level of 95% were chosen for an infinite population size [36]. To ensure sufficient representation, the minimum sample size for the pre-survey was set at 59 valid respondents from each of the 193 member states of the United Nations. To account for potential challenges in conducting face-to-face interviews and the risk of missing responses without interviewer guidance and support [37], the sample size was increased by 10%. From a statistical modeling perspective, having a minimum of 10 participants with complete responses per independent variable enables regression analyses with a minimum probability level (p-value) of 0.05 [38]. In this study, the complete data of 16,866 participants (79.9% of the dataset's total of 21,106 respondents) were extracted for analysis.

### Participant recruitment

During the first wave of the COVID-19 pandemic in 2020, participants were recruited using a respondent-driven sampling approach. Initially, 45 members of the MEHEWE Study Group (www.mehewe.org) reached out to potential participants, who were then asked to share the survey link with their contacts worldwide, aiding in the recruitment process. The survey link was also disseminated through various channels, including social media groups (Facebook, Twitter, and Instagram), network email lists, and WhatsApp groups. Comprehensive information about the survey implementation and data collection tools can be found in previous studies [39–43]. Details on the sample had also been reported [44, 45]. Participants had to check a box to indicate consent to participate to be able to continue with the survey. Also, objectives of the study, duration needed to answer the questionnaire (11 min) and statement mentioning voluntary participation was added before initiating the survey. No identifier data were collected from respondents.

### Data collection tool

The developed tool underwent rigorous assessment to evaluate its validity, dimensionality, and reliability. Initially developed in English, it was subsequently translated into French, Spanish, Arabic, and Portuguese to ensure wider accessibility. The study questionnaire achieved an overall content validation index of 0.83, indicating a high level of agreement among experts regarding the content validity of the instrument. Detailed information on the validation process for the data collection tool has been previously published [39].

### Dependent variables

The study examined self-reported adherence to COVID-19 preventive measures during the initial wave of the pandemic, including the wearing of face masks, frequent hand washing or sanitizing, practicing physical distancing, and working remotely. Participants were instructed to indicate their adoption of each measure by checking the corresponding box(es). A checked box indicated the individual had implemented the specified measure during the pandemic. The survey questions used for assessing adherence were derived from the pandemic stress index, which achieved a content validity index of 0.90 [39]. Furthermore, the internal consistency of the adherence assessment tool was found to be high, as indicated by a Cronbach's alpha value of 0.80 [39].

### Independent variables

The study considered several independent variables: age, sex at birth (male or female), the highest level of education achieved (none, primary, secondary, college/university), and employment status (retirees, students, not employed, employed). Additionally, participants were asked to indicate if they were living with any chronic disease from a provided list. Selecting the checkbox next to a specific disease indicated that the respondent was living with that condition. The list of diseases was designed to categorize respondents into three risk levels for severe COVID-19: little or no risk, potential risk, and high risk [46]. The section of the survey collecting this data achieved a content validity index of 0.83 [39].

### Effect modifier/ moderator

Country income level was treated as an effect modifier. Data about country income level was obtained from publicly available data from the 2019 World Bank Data on country classification by Gross National Income [47]. The income level of a country can significantly impact policy formulation and the capacity of healthcare systems to respond to the COVID-19 pandemic [48]. Based on their income levels, countries were classified into four categories: low-income countries (LICs) with a gross national income (GNI) per capita ≤ 1035 USD in 2019, lower middle-income countries (LMICs) with a GNI between 1036 and 4045 USD, upper middle-income countries (UMICs) with a GNI between 4046 and 12,535 USD, and high-income countries (HICs) with a GNI ≥ 12,536 USD.

### Data analysis

The raw data were downloaded, cleaned, and imported into SPSS version 23.0 (IBM Corp., Armonk, N.Y., USA) for analysis. Four multivariable logistic regression models were constructed to examine the associations between each dependent variable and the independent variables. To evaluate the moderating effect of country income level on the relationship between the independent variables and the four dependent variables, we calculated the *p*-value for the interaction effect between country income level and each independent variable. We then divided the sample based on country income level and conducted separate regression analyses with the independent variables. For all models, the adjusted odds ratios (AORs), 95% confidence intervals (CIs), and p-values were calculated. Statistical significance was defined as *p* < 0.05.

## Results

In Table 1, the dataset consisted of 16,866 respondents. Among them, 11,464 (68.0%) reported adhering to physical distancing, 12,634 (74.9%) reported wearing masks or face coverings, 12,336 (73.1%) reported frequent hand washing or sanitizing, and 5,646 (33.5%) reported working remotely. Most respondents were from lower middle-income countries (LMICs) (53.9%), identified as female (62.3%), had a college/university education (78.1%), were employed (58.0%), and were categorized as having little or no risk for severe COVID-19 (77.9%)

**Table 1:** The socio-demographic profile of respondents who adhere to COVID-19 preventive measures during the first wave of the COVID-19 pandemic (*N* = 16,866)

Variables	Total *N* = 16,866 n (%)	Physical distancing		Wearing mask or face covering		Washing or sanitizing hands		Work remotely	
		Yes *N* = 11,464 (68.0%) n (%)	No *N* = 5402 (32.0%) n (%)	Yes *N* = 12,634 (74.9%) n (%)	No *N* = 4232 (25.1%) n (%)	Yes *N* = 12,336 (73.1%) n (%)	No *N* = 4530 (26.9%) n (%)	Yes *N* = 5646 (33.5%) n (%)	No *N* = 11,220 (66.5%) n (%)
**Economic region**									
LIC	404 (2.4)	254 (62.9)	150 (37.1)	277 (68.6)	127 (31.4)	301 (74.5)	103 (25.5)	133 (32.9)	271 (67.1)
LMIC	8935 (53.9)	5488 (61.4)	3447 (38.6)	6341 (71.0)	2594 (29.0)	6115 (68.4)	2820 (34.6)	2508 (28.1)	6427 (71.9)
UMIC	3449 (20.4)	2630 (76.3)	819 (23.7)	2844 (82.5)	605 (17.5)	2710 (78.6)	739 (21.4)	1236 (35.8)	2213 (64.2)
HIC	4078 (24.2)	3092 (75.8)	986 (24.2)	3172 (77.8)	906 (22.2)	3210 (78.7)	868 (21.3)	1769 (43.4)	2309 (56.6)
**Age**	35.3 (12.9)	36.5 (13.0)	32.6 (12.3)	36.0 (13.0)	33.1 (12.4)	36.1 (13.0)	33.1 (12.4)	36.5 (12.1)	34.7 (13.3)
**Sex at birth**									
Male	6366 (37.7)	4402 (69.1)	1964 (30.9)	4720 (74.1)	1646 (25.9)	4492 (70.6)	1874 (29.4)	2120 (33.3)	4246 (66.7)
Female	10,500 (62.3)	7062 (67.3)	3438 (32.7)	7914 (75.4)	2586 (24.6)	7844 (74.7)	2656 (25.3)	3526 (33.6)	6974 (66.4)
**Level of education**									
No formal education	309 (1.8)	61 (19.7)	248 (80.3)	156 (50.5)	153 (49.5)	169 (54.7)	140 (45.3)	51 (16.5)	258 (83.5)
Primary	398 (2.4)	125 (31.4)	273 (68.6)	192 (48.2)	206 (51.8)	186 (46.7)	212 (53.3)	58 (14.6)	340 (85.4)
Secondary	2980 (17.7)	1779 (59.7)	1201 (40.3)	2136 (71.7)	844 (28.3)	2081 (69.8)	899 (30.2)	648 (21.7)	2332 (78.3)
College/university	13,179 (78.1)	9499 (72.1)	3680 (27.9)	10,150 (77.0)	3029 (23.0)	9900 (75.1)	3279 (24.9)	4889 (37.1)	8290 (62.9)
**Employment status**									
Retiree	693 (4.1)	499 (72.0)	194 (28.0)	536 (77.3)	157 (22.7)	525 (75.8)	168 (24.2)	78 (11.3)	615 (88.7)
Student	3750 (22.2)	2343 (62.5)	1407 (37.5)	2644 (70.5)	1106 (29.5)	2564 (68.4)	1186 (31.6)	1097 (29.3)	2653 (70.7)
Employed	9787 (58.0)	7155 (73.1)	2632 (26.9)	7609 (77.7)	2178 (22.3)	7423 (75.8)	2364 (24.2)	4006 (40.9)	5781 (59.1)
Unemployed	2636 (15.6)	1467 (55.7)	1169 (44.3)	1845 (70.0)	791 (30.0)	1824 (69.2)	812 (30.8)	465 (17.6)	2171 (82.4)
**Risk for severe COVID-19**									
Little or no risk	13,134 (77.9)	8842 (67.3)	4292 (32.7)	9682 (73.7)	3452 (26.3)	9432 (71.8)	3702 (28.2)	4292 (32.7)	8842 (67.3)
Might be at increased risk	2791 (16.5)	1992 (71.4)	799 (28.6)	2246 (80.5)	545 (19.5)	2204 (79.0)	587 (21.0)	1026 (36.8)	1765 (63.2)
At increased risk	941 (5.6)	630 (67.0)	311 (33.0)	706 (75.0)	235 (25.0)	700 (74.4)	241 (25.6)	328 (34.9)	613 (65.1)

Table 2 presents the findings from adjusted multivariate regressions analyzing the associations between the dependent variables and independent variables. Regarding the practice of physical distancing, individuals who may be at increased risk for severe COVID-19 had higher odds of adherence (AOR: 1.202; *p* < 0.001) compared to those with little or no risk. Age was also associated with increased odds of practicing physical distancing (AOR: 1.025; *p* < 0.001). In terms of education, respondents with no formal education (AOR: 0.134; *p* < 0.001), primary education (AOR: 0.204; *p* < 0.001), and secondary education (AOR: 0.660; *p* < 0.001) had lower odds of physical distancing compared to those with college/university education. Additionally, retirees (AOR: 0.485; *p* < 0.001) and the unemployed (AOR: 0.655; *p* < 0.001) had significantly lower odds of practicing physical distancing compared to the employed.

**Table 2:** Adjusted multivariate regression analysis showing socio-demographic factors associated with adherence to COVID-19 preventive measures during the first wave of the COVID-19 pandemic (*N* = 16,866)

Variables	Physical distancing	Wearing mask or face covering	Washing or sanitizing hands	Work remotely
	AOR; 95% CI; *p* value	AOR; 95% CI; *p* value	AOR; 95% CI; *p* value	AOR; 95% CI; *p* value
**Country income level**				
LICs	0.487; 0.392–0.606; *p* < 0.001	0.596; 0.476–0.747; *p* < 0.001	0.768; 0.605–0.974; *p* = 0.030	0.579; 0.464–0.723; *p* < 0.001
LMICs	0.621; 0.569–0.677; *p* < 0.001	0.817; 0.747–0.893; *p* < 0.001	0.678; 0.619–0.741; *p* < 0.001	0.565; 0.521–0.613; *p* < 0.001
UMICs	1.071; 0.961–1.194;* p* = 0.217	1.392; 1.239–1.564; *p* < 0.001	1.010; 0.902–1.130; *p* = 0.865	0.804; 0.729–0.885; *p* < 0.001
HICs	1.000	1.000	1.000	1.000
**Sex at birth**				
Male	1.015; 0.946–1.090; *p* = 0.678	0.896; 0.832–0.966; *p* = 0.004	0.769; 0.715–0.827; *p* < 0.001	0.874; 0.816–0.938; *p* < 0.001
Female	1.000	1.000	1.000	1.000
**Age**	1.025; 1.021–1.028; *p* < 0.001	1.016; 1.012–1.020; *p* < 0.001	1.018; 1.014–1.022; *p* < 0.001	1.013; 1.010–1.017; *p* < 0.001
**Level of education**				
No formal education	0.134; 0.100–0.180; *p* < 0.001	0.360; 0.284–0.456; *p* < 0.001	0.471; 0.372–0.598; *p* < 0.001	0.583; 0.425–0.800; *p* = 0.001
Primary	0.204; 0.163–0.254; *p* < 0.001	0.292; 0.237–0.359; *p* < 0.001	0.309; 0.251–0.380; *p* < 0.001	0.382; 0.286–0.510; *p* < 0.001
Secondary	0.660; 0.604–0.720; *p* < 0.001	0.843; 0.767–0.927; *p* < 0.001	0.858; 0.782–0.942; *p* < 0.001	0.555; 0.502–0.613; *p* < 0.001
College/university	1.000	1.000	1.000	1.000
**Employment status**				
Retiree	0.485; 0.396–0.595; *p* < 0.001	0.557; 0.450–0.691; *p* < 0.001	0.562; 0.456–0.693; *p* < 0.001	0.119; 0.092–0.155; *p* < 0.001
Student	0.943; 0.853–1.043; *p* = 0.255	0.872; 0.784–0.969; *p* = 0.011	0.885; 0.798–0.981; *p* = 0.020	0.823; 0.745–0.909; *p* < 0.001
Employed	1.000	1.000	1.000	1.000
Unemployed	0.655; 0.594–0.722; *p* < 0.001	0.823; 0.741–0.913; *p* < 0.001	0.854; 0.771–0.946; *p* = 0.003	0.369; 0.329–0.413; *p* < 0.001
**Risk for severe COVID-19**				
Little or no risk	1.000	1.000	1.000	1.000
Might be at increased risk	1.202; 1.092–1.323; *p* < 0.001	1.471; 1.325–1.634; *p* < 0.001	1.449; 1.309–1.604; *p* < 0.001	1.268; 1.159–1.388; *p* < 0.001
At increased risk	0.895; 0.765–1.046; *p* = 0.163	1.029; 0.875–1.211; *p* = 0.727	1.069; 0.910–1.255; *p* = 0.418	1.159; 0.995–1.350; *p* = 0.058

For the use of face masks or face coverings, respondents at increased risk of severe COVID-19 had higher odds of wearing them (AOR: 1.471; *p* < 0.001) compared to those with little or no risk. Older age was also associated with increased odds of wearing masks or face coverings (AOR: 1.016; *p* < 0.001). Males had lower odds of wearing face masks or coverings compared to females (AOR: 0.896; *p* = 0.004). Furthermore, respondents with no formal education (AOR: 0.360; *p* < 0.001), primary education (AOR: 0.296; p < 0.001), and secondary education (AOR: 0.843; *p* < 0.001) had lower odds of wearing masks or face coverings compared to college/university graduates. Retirees (AOR: 0.557; *p* = 0.035), students (AOR: 0.892; *p* = 0.011), and the unemployed (AOR: 0.823; *p* < 0.001) had significantly lower odds of wearing masks or face coverings compared to the employed.

Regarding frequent hand washing or sanitizing, respondents who might be at increased risk of severe COVID-19 had higher odds of engaging in this practice (AOR: 1.499; *p* < 0.001) compared to those with little or no risk. Age was also associated with increased odds of frequent hand washing or sanitizing (AOR: 1.018; *p* < 0.001). Males had lower odds of practicing frequent hand hygiene compared to females (AOR: 0.769; *p* < 0.001). Respondents with no formal education (AOR: 0.471; *p* < 0.001), primary education (AOR: 0.309; *p* < 0.001), and secondary education (AOR: 0.858; *p* < 0.001) had lower odds of frequent hand washing or sanitizing compared to college/university graduates. Additionally, retirees (AOR: 0.562; *p* < 0.001), students (AOR: 0.885; *p* = 0.020), and the unemployed (AOR: 0.854; *p* = 0.003) had lower odds of practicing frequent hand hygiene compared to the employed.

Regarding remote work, individuals who might be at increased risk of severe COVID-19 had higher odds of working remotely (AOR: 1.268; *p* = 0.002) compared to those with little or no risk. Age was also associated with increased odds of remote work (AOR: 1.013; *p* < 0.001). Males had lower odds of working remotely compared to females (AOR: 0.874; *p* < 0.001). Respondents with no formal education (AOR: 0.583; *p* < 0.001), primary education (AOR: 0.382; *p* < 0.001), and secondary education (AOR: 0.555; *p* < 0.001) had lower odds of working remotely compared to college/university graduates. Additionally, retirees (AOR: 0.119; *p* < 0.001), students (AOR: 0.823; *p* < 0.001), and the unemployed (AOR: 0.369; *p* < 0.001) had significantly lower odds of working remotely compared to the employed.

Table 3 shows that the practice of physical distancing, country income level significantly influenced the association between all factors and adherence. Specifically, the effect of sex was significant, with males having higher odds of physical distancing than females in LMICs, but lower odds in UMICs and HICs. Older age was associated with higher odds of physical distancing in LICs, LMICs, and HICs, but this association was not statistically significant in UMICs. Participants with lower education status had significantly lower odds of practicing physical distancing compared to those with college/university education only in LMICs and UMICs. Additionally, retirees and the unemployed had significantly higher odds of practicing physical distancing compared to the employed only in UMICs and HICs. Participants who might be at increased risk for severe COVID-19 had significantly higher odds of practicing physical distancing only in UMICs and HICs.

**Table 3:** Moderating effect of country income level on the association between adherence to COVID-19 preventive measures and socio-demographic factors (*N* = 16,866)

Variables	AOR; 95% CI; p value				*P* value of interaction
	Low-income countries	Low middle-income countries	Upper middle-income countries	High-income countries	
**Physical distancing**					
Sex at birth					
Male	1.420 (0.903, 2.233); 0.129	1.181 (1.074, 1.298); 0.001	0.772 (0.652, 0.914); 0.003	0.729 (0.628, 0.847); < 0.001	< 0.001
Female	1.00	1.00	1.00	1.00	
Age	1.025 (1.001, 1.05); 0.040	1.029 (1.024, 1.035); < 0.001	1.010 (1.002, 1.018); 0.141	1.023 (1.016, 1.030); < 0.001	0.024
Level of education					
No formal education	0 (0, -); 1.00	0.124 (0.090, 0.171); < 0.001	0.239 (0.064, 0.899); 0.034	0.346 (0.090, 1.323); 0.121	0.001
Primary	-	0.172 (0.132, 0.224); < 0.001	0.318 (0.169, 0.600); < 0.001	0.363 (0.191, 0.689); 0.002	
Secondary	0.961 (0.370, 2.494); 0.935	0.566 (0.504, 0.637); < 0.001	0.769 (0.624, 0.949); 0.014	0.875 (0.718, 1.067); 0.187	
College/university	1.00	1.00	1.00	1.00	
Employment status					
Retiree	1.051 (0.142, 7.812); 0.961	1.402 (0.938, 2.097); 0.099	2.276 (1.478, 3.507); < 0.001	2.614 (1.617, 4.226); < 0.001	< 0.001
Student	1.835 (0.294, 11.451); 0.163	1.875 (1.304, 2.696); 0.001	1.834 (1.315, 2.557); < 0.001	1.703 (1.124, 2.580); 0.012	
Employed	1.00	1.00	1.00	1.00	
Unemployed	0.757 (0.115, 4.993); 0.773	1.068 (0.728, 1.567); 0.737	1.481 (1.012, 2.168); 0.043	1.651 (1.036, 2.631); 0.035	
Risk for severe COVID-19					
Little or no risk	1.00	1.00	1.00	1.00	< 0.001
Might be at increased risk	1.240 (0.654, 2.348); 0.510	1.037 (0.910, 1.181); 0.586	1.580 (1.257, 1.987); < 0.001	1.435 (1.175, 1.753); < 0.001	
At increased risk	1.266 (0.460, 3.485); 0.648	0.728 (0.582, 0.911); 0.006	1.554 (1.076, 2.246); 0.019	1.004 (0.746, 1.351); 0.980	
**Wearing mask or face covering**					
Sex at birth					
Male	1.041 (0.649, 1.671); 0.868	0.963 (0.872, 1.063); 0.457	0.626 (0.519, 0.754); < 0.001	0.829 (0.710, 0.967); 0.017	0.001
Female	1.00	1.00	1.00	1.00	
Age	1.031 (1.005, 1.057); 0.019	1.017 (1.011, 1.022); < 0.001	1.01 (1.001, 1.02); 0.038	1.017 (1.010, 1.024); < 0.001	0.745
Level of education					
No formal education	0 (0, -); 1.00	0.375 (0.292, 0.482); < 0.001	0.172 (0.045, 0.652); 0.010	0.190 (0.050, 0.719); 0.014	0.901
Primary	-	0.299 (0.236, 0.377); < 0.0001	0.296 (0.153, 0.570); < 0.001	0.237 (0.125, 0.447); < 0.001	
Secondary	0.776 (0.298, 2.020); 0.603	0.834 (0.737, 0.943); 0.004	0.930 (0.729, 1.186); 0.559	0.852 (0.694, 1.045); 0.124	
College/university	1.00	1.00	1.00	1.00	
Employment status			2.259 (1.387, 3.680); 0.001		< 0.001
Retiree	0.716 (0.065, 7.848); 0.784	1.055 (0.701, 1.587); 0.798	1.748 (1.199, 2.547); 0.0004	2.901 (1.786, 4.714); < 0.001	
Student	1.191 (0.125, 11.320); 0.879	1.742 (1.207, 2.514); 0.0003	1.00	1.566 (1.035, 2.369); 0.034	
Employed	1.00	1.00	1.277 (0.832, 1.961); 0.263	1.00	
Unemployed	0.470 (0.048, 4.640); 0.518	1.289 (0.873, 1.903); 0.202		1.970 (1.228, 3.160); 0.005	
Risk for severe COVID-19					
Little or no risk	1.00	1.00	1.00	1.00	0.008
Might be at increased risk	1.556 (0.772, 3.136); 0.217	1.410 (1.225, 1.623); < 0.001	2.232 (1.678, 2.968); < 0.001	1.312 (1.072, 1.605); 0.008	
At increased risk	0.621 (0.238, 1.618); 0.329	0.892 (0.713, 1.116); 0.318	1.642 (1.083, 2.489); 0.020	1.217 (0.889, 1.665); 0.220	
**Washing or sanitizing hands**					
Sex at birth					
Male	0.910 (0.555, 1.490); 0.707	0.842 (0.765, 0.927); < 0.001	0.591 (0.497, 0.702); < 0.001	0.644 (0.551, 0.752); < 0.001	0.001
Female	1.00	1.00	1.00	1.00	
Age	1.029 (1.002, 1.056); 0.032	1.022 (1.016, 1.028); < 0.001	1.01 (0.998, 1.014); 0.143	1.017 (1.010, 1.025); < 0.000	0.170
Level of education					
No formal education	0 (0, -); 1.00	0.462 (0.360, 0.594); < 0.001	0.342 (0.090, 1.291); 0.113	0.876 (0.177, 4.349); 0.872	0.698
Primary	-	0.317 (0.251, 0.401); < 0.001	0.257 (0.136, 0.485); < 0.001	0.284 (0.150, 0.539); < 0.001	
Secondary	0.603 (0.233, 1.562); 0.298	0.831 (0.737, 0.937); 0.003	0.867 (0.695, 1.083); 0.208	1.043 (0.840, 1.294); 0.703	
College/university	1.00	1.00	1.00	1.00	
Employment status					
Retiree	1.374 (0.122, 15.470); 0.437	1.178 (0.786, 1.764); 0.427	1.910 (1.218, 2.994); 0.005	2.380 (1.438, 3.938); 0.001	< 0.001
Student	1.337 (0.140, 12.792); 0.801	1.756 (1.220, 2.528); 0.002	1.593 (1.126, 2.253); 0.009	1.463 (0.947, 2.258); 0.086	
Employed	1.00	1.00	1.00	1.00	
Unemployed	0.825 (0.082, 8.293); 0.870	1.408 (0.957, 2.072); 0.082	1.191 (0.802, 1.769); 0.387	1.833 (1.117, 3.008); 0.016	
Risk for severe COVID-19					0.214
Little or no risk	1.00	1.00	1.00	1.00	
Might be at increased risk	1.285 (0.620, 2.665); 0.500	1.430 (1.247, 1.640); < 0.001	1.585 (1.249, 2.013); < 0.001	1.473 (1.192, 1.821); < 0.001	
At increased risk	0.633 (0.236, 1.699); 0.364	0.991 (0.793, 1.239); 0.938	1.792 (1.206, 2.661); 0.004	1.048 (0.771, 1.425); 0.765	
**Work remotely**					
Sex at birth					
Male	0.740 (0.468,1.172); 0.199	0.936 (0.849, 1.032); 0.187	0.857 (0.730, 1.007); 0.060	0.759 (0.662, 0.870); < 0.001	0.077
Female	1.00	1.00	1.00	1.00	
Age	1.027 (1.003, 1.052); 0.029	1.015 (1.010, 1.020); < 0.001	1.012 (1.005, 1.019); 0.001	1.016 (1.010, 1.022); < 0.001	0.088
Level of education					
No formal education	0 (0, -); 1.00	0.511 (0.369, 0.707); < 0.001	0.173 (0.021, 1.421); 0.102	0.309 (0.061, 1.566); 0.156	0.021
Primary	-	0.418 (0.304, 0.576); < 0.001	0.106 (0.025, 0.449); 0.002	0.343 (0.158, 0.742); 0.007	
Secondary	0.238 (0.052, 1.080); 0.063	0.659 (0.574, 0.756); < 0.001	0.428 (0.340, 0.539); <0.001	0.491 (0.407, 0.594); < 0.001	
College/university	1.00	1.00	1.00	1.00	
Employment status					
Retiree	0.797 (0.112, 5.657); 0.821	3.678 (2.218, 6.100); < 0.001	8.290 (5.050, 13.610); < 0.001	13.148 (7.577, 22.813); < 0.001	< 0.001
Student	1.317 (0.234, 7.413); 0.755	5.026 (3.140, 8.043); < 0.001	10.873 (7.149, 16.538); < 0.001	11.189 (6.743, 18.566); < 0.001	
Employed	1.00	1.00	1.00	1.00	
Unemployed	0.238 (0.036, 1.569); 0.136	3.102 (1.896, 5.076); < 0.001	1.710 (1.034, 2.829); 0.037	2.002 (1.138, 3.523); 0.016	
Risk for severe COVID-19					
Little or no risk	1.00	1.00	1.00	1.00	0.086
Might be at increased risk	1.289 (0.684, 2.429); 0.433	1.139 (0.998, 1.300); 0.053	1.246 (1.016, 1.529); 0.034	1.563 (1.320, 1.852); < 0.001	
At increased risk	0. 677 (0.268, 1.716); 0.411	1.087 (0.863, 1.370); 0.478	1.129 (0.798, 1.598); 0.492	1.358 (1.037, 1.777); 0.026	

For wearing face masks or coverings, the interactions with sex, employment status, and risk for severe COVID-19 were significant. Males had significantly lower odds of wearing a face mask compared to females only in UMICs and HICs. Retirees and students had significantly higher odds of wearing a face mask compared to the employed in UMICs and HICs. Only in HICs did unemployed participants have significantly higher odds of wearing a face mask compared to the employed. Participants who might be at increased risk for severe COVID-19 had significantly higher odds of wearing a face mask in LMICs, UMICs, and HICs.

Country income level also moderated the association between frequent hand washing or sanitization and sex and employment status. Males had significantly lower odds compared to females in LMICs, UMICs, and HICs. Students had significantly higher odds of sanitizing their hands frequently in LMICs and UMICs, but not in LICs or HICs, when compared to employed participants. Unemployed participants had significantly higher odds of sanitizing their hands only in HICs, whereas the associations in LICs, LMICs, and UMICs were statistically significant compared to employed participants.

The table also shows that country income level significantly modified the association between working remotely and educational level and employment status. Participants with primary or secondary education had significantly lower odds of working remotely than those with college/university education in LMICs, UMICs, or HICs, but not in LICs. Additionally, retirees, students, and unemployed individuals had significantly higher odds of working remotely than employed participants in LMICs, UMICs, and HICs, but not in LICs.

## Discussion

The results of the study indicate that older individuals, those who are employed, have a college/university education, and have a higher risk for severe COVID-19, are more likely to adhere to all the studied COVID-19 preventive measures. While there was no significant difference in physical distancing behavior between male and female respondents, it was observed that male respondents appeared to be less likely to adhere to the other three COVID-19 preventive measures.

The associations between age and adherence to physical distancing measures seem to be influenced by the income level of the county. The observed higher likelihood of adhering to physical distancing with increased age was not observed in LIMCs. Additionally, while males were generally less likely than females to adhere to wearing face masks and sanitizing hands, this gender difference was not evident in LICs. Similarly, the associations between wearing face masks, physical distancing, hand sanitizing, and working remotely based on employment status were not observed in LICs. However, these associations held to varying degrees among HICs, UMICs, and LMICs. Although higher education levels, such as college/university education, were generally associated with a greater likelihood of adhering to all preventive measures, this relationship was not observed in LICs. Lastly, individuals at an increased risk of severe COVID-19 seem more likely to practice physical distancing and wear face masks in HICs and UMICs but were only more likely to wear face masks in LMICs.

These study findings contribute to the global data on factors associated with adherence to COVID-19 preventive measures. There are variations between countries in relation to the specific factors associated with the adherence to the COVID-19 preventive measures. For example, the use of hand hygiene was high in regions in the Democratic Republic of Congo that had experienced of Ebola [49]. Also, the use of facemasks was high in South Korea that had experienced MERS and had health concerns about the effects of particulate matter [50]. The present study provides information generated from a large sample of participants from different countries that can allow inferences to inform global actions.

The generalisability of the study is, however, limited by some study methods. These include the use of a non-probability sampling technique that increases the risk of excluding participants based on their inability to access the internet, use smartphones or due to language barriers. This sampling bias skewed the data towards populations with higher education levels [51, 52]. This was because data was collected electronically during the first wave of the pandemic when movement was highly restricted in many countries. Thus, the ability to use a probability sampling technique for data collection was limited [53, 54]. Nevertheless, considering the urgency for information during the COVID-19 pandemic, the ethical imperative to generate new information during the pandemic, and the restrictions on physical contact, the use of an online survey was a suitable approach [54]. It is important, therefore, to interpret our findings within these limitations and to take into consideration that what is reported in the study cannot be interpreted as population prevalence estimates. The cross-sectional design also limits the ability to make causal-effect inferences from the study findings. Furthermore, respondents had to self-report their health status by responding to a single question. This may be liable to response bias. Despite these limitations, the study highlights findings that provide a wealth of data relevant to the future pandemics of the magnitude of COVID-19.

The findings of the study indicate that individuals with lower educational status, younger age, male gender, retired or unemployed status, or studying were less likely to adhere to COVID-19 preventive measures. These observations align with prior studies [49, 55–59]. However, the associations between these factors and adherence were influenced by the country of residence, with greater variability in adherence levels observed in HICs and UMICs compared to LMICs and LICs. For instance, in HICs, UMICs, and LICs, adherence to physical distancing was more likely to occur with increased age, which may be attributed to older individuals having higher risk factors for COVID-19 and being more aware of preventive measures [60, 61]. Adherence may also have been lower in people with younger age because of the adduced lower risk of contracting COVID-19 among adolescents and public health messages had focused mainly on adults [62].

Like other studies, we also observed that females exhibited better adherence to COVID-19 preventive behaviors compared to males [63–65]. This gender difference may be due to females perceiving COVID-19 as a serious health problem with severe consequences [56]. Additionally, women's higher risk aversion tendencies [65–67] may contribute to this observed difference. These findings may partly explain the higher COVID-19 mortality rates among men, although other social and biological factors are also involved [68–70]. Bridging the gender gap can be facilitated by firsthand experiences of the pandemic and living in households rather than living alone [71]. Gender differences in the use of preventive measures were observed in HICs, UMICs, and LMICs but not in LICs. This finding emphasizes the importance of gender-sensitive messaging in COVID-19 prevention and contributes to the understanding of gender disparities in the impact of COVID-19 and health outcomes.

Furthermore, individuals who perceived themselves to be at risk of severe COVID-19 were more likely to adhere to preventive measures. Interestingly, those who were at risk did not demonstrate higher adherence, and this improved adherence was only observed in HICs and UMICs. Many LICs and LMICs faced challenges in making masks and hand sanitizers readily available to their citizens during the pandemic, primarily due to issues with affordability and supply [72]. However, LICs and LMICs also have a higher proportion of younger populations who were poorly adhering to COVID-19 preventive measures when compared to adults. A prior meta-analysis indicated that young people were least likely to comply with physical distancing, face masking. Their hand hygiene practice was the best [73]. Young people were also less likely to work remotely in LICs and LMICs because of the poor online schooling opportunities accessible to them [74]. Cultural barriers and poorer community perception of danger in lower-income countries may also play a role [75, 76]. Additionally, the higher number of COVID-19 deaths in HICs may contribute to better adherence to preventive measures, in line with the health belief model's proposition that risk perception influences health-related behaviors. Further research is needed to explore this finding.

Finally, we observed fewer sociodemographic differences in adherence levels in LICs compared to HICs and UMICs, and to a lesser extent in LMICs. The significant sociodemographic disparities observed in HICs and UMICs, compared to LMICs and LICs, suggest that macro-level factors may have influenced individual responses to the pandemic. Country income level plays a substantial role in the observed disparities in COVID-19 response [77, 78]. However, little is known about how these macro-level factors impact individual responses to the COVID-19 pandemic. Public policies that promote welfare during crises, such as family policies, employment policies, income support, social insurance policies, area-based initiatives, and education policies, may influence the adoption of COVID-19 preventive measures [79]. The study findings highlight the need to study the complex interrelationship between sociodemographic factors, adherence to COVID-19 preventive measures, and macro-level factors. The index of stringency in compliance with preventive measures indicates better compliance in HICs and UMICs compared to LMICs and LICs, respectively [80].

In conclusion, we observed that younger individuals, males, those with lower educational status, and those without chronic disease related elevated risk for severe COVID-19 were less likely adhere to COVID-19 preventive measures, and that country income levels moderated these associations. The study findings suggests that although micro-social determinants of adherence to COVID-19 preventive measures may be influenced by pre-existing health behavior determinants, the country income level appears to be a macro-social factor that moderates the micro-social determinants of adherence to COVID-19 preventive measures. Understanding how and why country income level moderates the socio-demographic determinants of adherence to preventive measures is crucial.

## Data Availability

The datasets used and/or analysed during the current study are available from the corresponding author upon reasonable request.

## Abbreviations

AOR: Adjusted Odds Ratio
CI: Confidence Interval
COVID-19: Coronavirus infectious disease 2019
GNI: Gross national income
HICs: High‐income countries
ICT: Information and communications technology
LICs: Low‐income countries
LMICs: Lower middle‐income countries
SD: Standard Deviation
UMICs: Upper middle‐income countries
USD: United States Dollars
